# 2,5-Bis(4-methyl­phen­yl)-4-oxopenta­noic acid

**DOI:** 10.1107/S1600536810037323

**Published:** 2010-09-25

**Authors:** Jun Wang, Xiaomei Zhuang, Yong Hou

**Affiliations:** a Zhongshan Polytechnic, Zhongshan, Guangdong 528404, People’s Republic of China

## Abstract

The title compound, C_19_H_20_O_3_, was obtained from 1,4-bis­(4-methyl­phen­yl)but-3-yn-2-one in the presence of carbon monoxide by Ni(CN)_2_ catalysis in a basic aqueous medium. Inter­molecular O—H⋯O hydrogen bonds lead to the formation of hydrogen-bonded carb­oxy­lic acid dimers [graph-set motif *R* ^2^_2_(8)]. Weak C—H⋯O hydrogen bonds between neighbouring dimers further extend the structure to give rise to a three-dimensional supra­molecular network.

## Related literature

For general background to transition metal-mediated carbonyl­ation reactions, see: Collins (1999 ▶); Arzoumanian *et al.* (1995 ▶). For a similar structure, see: Garcia-Gutierrez *et al.* (2004 ▶). For bond length values, see: Allen *et al.* (1987 ▶). For hydrogen-bonding motifs, see: Bernstein *et al.* (1995 ▶).
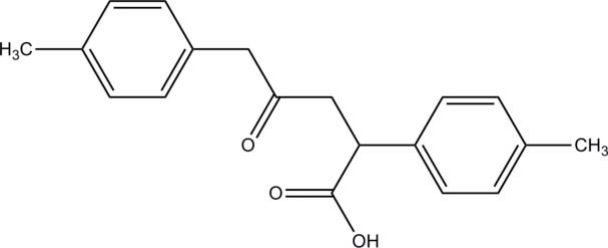


## Experimental

### 

#### Crystal data

C_19_H_20_O_3_
*M* *_r_* = 296.35
Monoclinic, 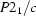

*a* = 11.846 (2) Å
*b* = 13.155 (3) Å
*c* = 11.755 (2) Å
β = 115.98 (3)°
*V* = 1646.7 (7) Å^3^
*Z* = 4
Mo *K*α radiation
μ = 0.08 mm^−1^
*T* = 293 K
0.25 × 0.22 × 0.19 mm

#### Data collection

Bruker APEXII area-detector diffractometer
12947 measured reflections
2956 independent reflections
1474 reflections with *I* > 2σ(*I*)
*R*_int_ = 0.062

#### Refinement

*R*[*F* ^2^ > 2σ(*F* ^2^)] = 0.050
*wR*(*F* ^2^) = 0.173
*S* = 1.01
2956 reflections
202 parameters
H-atom parameters constrained
Δρ_max_ = 0.24 e Å^−3^
Δρ_min_ = −0.18 e Å^−3^

### 

Data collection: *APEX2* (Bruker, 2004 ▶); cell refinement: *APEX2* and *SAINT* (Bruker, 2004 ▶); data reduction: *SAINT*; program(s) used to solve structure: *SHELXS97* (Sheldrick, 2008 ▶); program(s) used to refine structure: *SHELXL97* (Sheldrick, 2008 ▶); molecular graphics: *XP* in *SHELXTL* (Sheldrick, 2008 ▶); software used to prepare material for publication: *SHELXL97*.

## Supplementary Material

Crystal structure: contains datablocks I, global. DOI: 10.1107/S1600536810037323/zl2309sup1.cif

Structure factors: contains datablocks I. DOI: 10.1107/S1600536810037323/zl2309Isup2.hkl

Additional supplementary materials:  crystallographic information; 3D view; checkCIF report

## Figures and Tables

**Table 1:** Hydrogen-bond geometry (Å, °)

*D*—H⋯*A*	*D*—H	H⋯*A*	*D*⋯*A*	*D*—H⋯*A*
C17—H17⋯O1^i^	0.93	2.50	3.418 (4)	169
C15—H15⋯O2^ii^	0.93	2.56	3.452 (4)	160
O2—H2*A*⋯O3^iii^	0.82	1.83	2.638 (2)	169

Symmetry codes: (i) 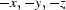; (ii) 
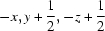
; (iii) 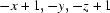.

**Fig. 1.**
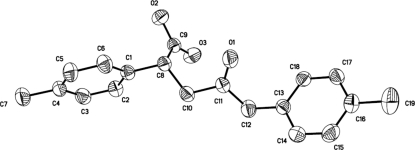
ORTEP represention of atom numbering diagram for the title compound, showing 30% probability displacement ellipsoids.

**Fig. 2.**
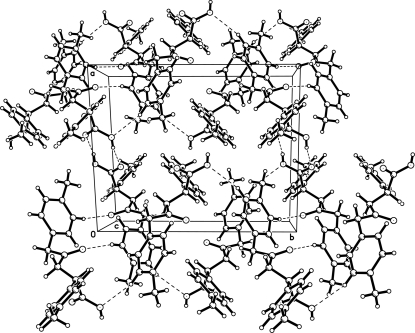
View of the three-dimensional structure of the title compound. H-bonds are shown as dashed lines.

## supplementary crystallographic information

### Comment

Transition-metal-mediated carbonylation reactions are of great research interest in recent years (Collins, 1999). Amongst the many metal-mediated syntheses used, catalysis by nickel cyanide in aqueous media under phase transfer conditions has attracted particular attention (Arzoumanian *et al.*, 1995) and numerous lactones and their hydrolysis products have been synthesized using this system. Herein, we chose 1,4-di(4-methylbenzyl)but-3-yn-2-one as a carbonylation substrate to be reacted in the presence of Ni(CN)_2_ and carbon monoxide in a biphasic toluene/basic aqueous medium to give the title compound.

The structure of the title compound is depicted in Fig. 1. The C—C bond lengths show normal values (Allen *et al.*, 1987), and the C—O and C═O bond lengths are comparable to those observed in simliar structures (Garcia-Gutierrez *et al.*, 2004). The molecules form dimers with neighboring molecules through O—H···O hydrogen bonding with an *R*^2^_2_(8) graph set motif (Bernstein *et al.*, 1995). These dimers are further linked by C—H···O hydrogen bonds (Table 1) to form a three-dimensional supramolecular network (Fig. 2).

### Experimental

A typical experiment was performed as follows: in a round-bottomed flask toluene (25 ml) and 1 M aqueous NaOH (10 ml) were degassed and saturated with CO under atmospheric pressure before Ni(CN)_2_.4H_2_O (1.0 mmol) and tetrabutylammonium bromide (0.3 mmol) were introduced, and the mixture was kept at room temperature overnight with stirring while CO was slowly (2–3 min) bubbled through the solution. To the yellow two-phase mixture was then added 10 mmol of 1,4-di(4-methylbenzyl)but-3-yn-2-one, and stirring and flow of CO at a flow rate of 3 ml min^-1^ were maintained for 5 h at 393 K. At the end of the reaction, ethyl ether (2 × 20 ml) was used to eliminate the impurities. The aqueous phase was acidified with diluted HCl at pH = 1. Ethyl ether (2 × 20 ml) was used to extract the product. The organic phase was dried over Na_2_SO_4_ and evaporated to obtain a yellow powder. During recrystallization, the yellow block crystals were obtained by slow evaporation of the solvent with a yield of 68%. m.p. 476–478 K; IR (KBr) cm^-1^: 3052, 2980, 2948, 1716, 1705, 1669, 1607, 1573, 1465, 1416, 1379, 1345, 1285, 1246, 1232, 1217, 1186, 1150, 1068, 1044, 995, 972, 850.

### Refinement

All H atoms attached to C and O atoms were fixed geometrically and treated as riding with C—H = 0.93 or 0.96 Å and O—H = 0.82 Å, and U_iso_(H) = 1.2U_eq_(C) and U_iso_(H) = 1.5U_eq_(O).

### Crystal data

**Table:**

C_19_H_20_O_3_	*F*(000) = 632
*M**_r_* = 296.35	*D*_x_ = 1.195 Mg m^−^^3^
Monoclinic, *P*2_1_/*c*	Mo *K*α radiation, λ = 0.71073 Å
Hall symbol: -P 2ybc	Cell parameters from 2279 reflections
*a* = 11.846 (2) Å	θ = 2.3–28.0°
*b* = 13.155 (3) Å	µ = 0.08 mm^−^^1^
*c* = 11.755 (2) Å	*T* = 293 K
β = 115.98 (3)°	Block, yellow
*V* = 1646.7 (7) Å^3^	0.25 × 0.22 × 0.19 mm
*Z* = 4	

### Data collection

**Table:**

Bruker APEXII area-detector diffractometer	1474 reflections with *I* > 2σ(*I*)
Radiation source: fine-focus sealed tube	*R*_int_ = 0.062
graphite	θ_max_ = 25.2°, θ_min_ = 3.1°
φ and ω scan	*h* = −14→14
12947 measured reflections	*k* = −15→15
2956 independent reflections	*l* = −14→14

### Refinement

**Table:**

Refinement on *F*^2^	Primary atom site location: structure-invariant direct methods
Least-squares matrix: full	Secondary atom site location: difference Fourier map
*R*[*F*^2^ > 2σ(*F*^2^)] = 0.050	Hydrogen site location: inferred from neighbouring sites
*wR*(*F*^2^) = 0.173	H-atom parameters constrained
*S* = 1.01	*w* = 1/[σ^2^(*F*_o_^2^) + (0.085*P*)^2^] where *P* = (*F*_o_^2^ + 2*F*_c_^2^)/3
2956 reflections	(Δ/σ)_max_ < 0.001
202 parameters	Δρ_max_ = 0.24 e Å^−^^3^
0 restraints	Δρ_min_ = −0.18 e Å^−^^3^

### Special details

**Table:**

Geometry. All esds (except the esd in the dihedral angle between two l.s. planes) are estimated using the full covariance matrix. The cell esds are taken into account individually in the estimation of esds in distances, angles and torsion angles; correlations between esds in cell parameters are only used when they are defined by crystal symmetry. An approximate (isotropic) treatment of cell esds is used for estimating esds involving l.s. planes.
Refinement. Refinement of F^2^ against ALL reflections. The weighted R-factor wR and goodness of fit S are based on F^2^, conventional R-factors R are based on F, with F set to zero for negative F^2^. The threshold expression of F^2^ > 2sigma(F^2^) is used only for calculating R-factors(gt) etc. and is not relevant to the choice of reflections for refinement. R-factors based on F^2^ are statistically about twice as large as those based on F, and R- factors based on ALL data will be even larger.

### Fractional atomic coordinates and isotropic or equivalent isotropic displacement parameters (Å^2^)

**Table:**

	*x*	*y*	*z*	*U*_iso_*/*U*_eq_	
C1	0.2848 (2)	0.06339 (18)	0.6978 (3)	0.0586 (7)	
C2	0.3764 (2)	0.1318 (2)	0.7725 (3)	0.0695 (8)	
H2	0.4156	0.1729	0.7360	0.083*	
C3	0.4101 (3)	0.1396 (2)	0.9004 (3)	0.0772 (9)	
H3	0.4722	0.1858	0.9485	0.093*	
C4	0.3548 (3)	0.0817 (2)	0.9587 (3)	0.0744 (8)	
C5	0.2638 (3)	0.0149 (3)	0.8837 (3)	0.0859 (9)	
H5	0.2239	−0.0254	0.9201	0.103*	
C6	0.2299 (3)	0.0057 (2)	0.7571 (3)	0.0758 (8)	
H6	0.1680	−0.0409	0.7099	0.091*	
C7	0.3949 (4)	0.0898 (3)	1.0989 (3)	0.1073 (12)	
H7A	0.4565	0.1427	1.1337	0.161*	
H7B	0.4304	0.0263	1.1388	0.161*	
H7C	0.3232	0.1057	1.1136	0.161*	
C8	0.2475 (2)	0.05361 (18)	0.5580 (2)	0.0568 (7)	
H8	0.1909	−0.0049	0.5272	0.068*	
C9	0.3590 (2)	0.0320 (2)	0.5332 (3)	0.0581 (7)	
C10	0.1766 (2)	0.1458 (2)	0.4817 (3)	0.0654 (7)	
H10A	0.1111	0.1638	0.5065	0.078*	
H10B	0.2341	0.2028	0.5021	0.078*	
C11	0.1187 (2)	0.1287 (2)	0.3424 (3)	0.0617 (7)	
C12	0.0992 (3)	0.2191 (2)	0.2583 (3)	0.0764 (8)	
H12A	0.0774	0.2768	0.2961	0.092*	
H12B	0.1783	0.2348	0.2563	0.092*	
C13	0.0008 (2)	0.20819 (18)	0.1251 (3)	0.0604 (7)	
C14	−0.1074 (3)	0.2653 (2)	0.0815 (3)	0.0827 (9)	
H14	−0.1173	0.3129	0.1349	0.099*	
C15	−0.2006 (3)	0.2536 (2)	−0.0386 (4)	0.0892 (10)	
H15	−0.2723	0.2937	−0.0646	0.107*	
C16	−0.1916 (3)	0.1845 (2)	−0.1219 (3)	0.0729 (8)	
C17	−0.0825 (3)	0.1291 (2)	−0.0809 (3)	0.0678 (8)	
H17	−0.0718	0.0832	−0.1355	0.081*	
C18	0.0115 (2)	0.14094 (19)	0.0405 (3)	0.0638 (7)	
H18	0.0843	0.1023	0.0659	0.077*	
C19	−0.2969 (3)	0.1693 (3)	−0.2526 (4)	0.1142 (13)	
H19A	−0.2762	0.1145	−0.2939	0.171*	
H19B	−0.3088	0.2305	−0.3010	0.171*	
H19C	−0.3729	0.1533	−0.2460	0.171*	
O1	0.0871 (2)	0.04379 (15)	0.3000 (2)	0.0947 (7)	
O2	0.41394 (17)	−0.05500 (14)	0.5781 (2)	0.0763 (6)	
H2A	0.4777	−0.0597	0.5674	0.114*	
O3	0.39643 (17)	0.08983 (15)	0.4773 (2)	0.0817 (7)	

### Atomic displacement parameters (Å^2^)

**Table:**

	*U*^11^	*U*^22^	*U*^33^	*U*^12^	*U*^13^	*U*^23^
C1	0.0534 (14)	0.0674 (16)	0.0574 (18)	0.0026 (11)	0.0266 (13)	−0.0028 (13)
C2	0.0662 (17)	0.0777 (18)	0.065 (2)	−0.0016 (13)	0.0293 (15)	−0.0019 (15)
C3	0.0674 (17)	0.088 (2)	0.064 (2)	0.0083 (14)	0.0171 (16)	−0.0111 (16)
C4	0.085 (2)	0.085 (2)	0.0540 (19)	0.0263 (16)	0.0306 (16)	0.0051 (16)
C5	0.105 (2)	0.097 (2)	0.073 (3)	0.0023 (18)	0.055 (2)	0.0100 (18)
C6	0.0804 (19)	0.087 (2)	0.069 (2)	−0.0133 (14)	0.0415 (17)	−0.0067 (16)
C7	0.132 (3)	0.126 (3)	0.060 (2)	0.048 (2)	0.039 (2)	0.010 (2)
C8	0.0558 (14)	0.0617 (14)	0.0549 (17)	−0.0005 (11)	0.0262 (12)	−0.0015 (12)
C9	0.0578 (15)	0.0647 (15)	0.0563 (17)	0.0003 (12)	0.0294 (13)	−0.0026 (13)
C10	0.0601 (15)	0.0771 (17)	0.0588 (19)	0.0056 (12)	0.0258 (13)	−0.0081 (13)
C11	0.0621 (15)	0.0671 (17)	0.0547 (18)	−0.0022 (12)	0.0245 (13)	−0.0054 (13)
C12	0.086 (2)	0.0749 (18)	0.064 (2)	−0.0135 (14)	0.0293 (16)	−0.0020 (15)
C13	0.0696 (16)	0.0574 (14)	0.0578 (18)	−0.0044 (12)	0.0312 (14)	0.0008 (13)
C14	0.095 (2)	0.084 (2)	0.074 (2)	0.0215 (16)	0.0407 (19)	−0.0013 (16)
C15	0.080 (2)	0.103 (2)	0.083 (3)	0.0331 (17)	0.0347 (19)	0.016 (2)
C16	0.0686 (17)	0.0887 (19)	0.060 (2)	−0.0010 (15)	0.0266 (15)	0.0068 (16)
C17	0.0831 (19)	0.0671 (17)	0.058 (2)	−0.0030 (14)	0.0355 (16)	−0.0034 (13)
C18	0.0693 (16)	0.0627 (16)	0.064 (2)	0.0077 (12)	0.0336 (15)	0.0056 (13)
C19	0.083 (2)	0.169 (4)	0.076 (3)	−0.010 (2)	0.021 (2)	0.009 (2)
O1	0.1269 (17)	0.0755 (14)	0.0615 (14)	−0.0101 (12)	0.0226 (12)	−0.0026 (11)
O2	0.0787 (13)	0.0734 (12)	0.0948 (17)	0.0158 (9)	0.0547 (12)	0.0179 (11)
O3	0.0835 (14)	0.0767 (12)	0.1091 (19)	0.0139 (9)	0.0646 (13)	0.0223 (12)

### Geometric parameters (Å, °)

**Table:**

C1—C6	1.372 (4)	C10—H10B	0.9700
C1—C2	1.387 (3)	C11—O1	1.214 (3)
C1—C8	1.508 (4)	C11—C12	1.498 (4)
C2—C3	1.380 (4)	C12—C13	1.494 (4)
C2—H2	0.9300	C12—H12A	0.9700
C3—C4	1.368 (4)	C12—H12B	0.9700
C3—H3	0.9300	C13—C14	1.376 (4)
C4—C5	1.371 (4)	C13—C18	1.378 (4)
C4—C7	1.506 (4)	C14—C15	1.367 (4)
C5—C6	1.366 (4)	C14—H14	0.9300
C5—H5	0.9300	C15—C16	1.375 (4)
C6—H6	0.9300	C15—H15	0.9300
C7—H7A	0.9600	C16—C17	1.374 (4)
C7—H7B	0.9600	C16—C19	1.509 (4)
C7—H7C	0.9600	C17—C18	1.381 (4)
C8—C9	1.499 (3)	C17—H17	0.9300
C8—C10	1.523 (3)	C18—H18	0.9300
C8—H8	0.9800	C19—H19A	0.9600
C9—O3	1.209 (3)	C19—H19B	0.9600
C9—O2	1.308 (3)	C19—H19C	0.9600
C10—C11	1.489 (4)	O2—H2A	0.8200
C10—H10A	0.9700		
			
C6—C1—C2	116.8 (3)	C8—C10—H10B	108.9
C6—C1—C8	121.9 (2)	H10A—C10—H10B	107.7
C2—C1—C8	121.3 (3)	O1—C11—C10	120.1 (3)
C3—C2—C1	120.7 (3)	O1—C11—C12	121.9 (3)
C3—C2—H2	119.6	C10—C11—C12	118.0 (2)
C1—C2—H2	119.6	C13—C12—C11	116.1 (2)
C4—C3—C2	122.0 (3)	C13—C12—H12A	108.3
C4—C3—H3	119.0	C11—C12—H12A	108.3
C2—C3—H3	119.0	C13—C12—H12B	108.3
C3—C4—C5	116.7 (3)	C11—C12—H12B	108.3
C3—C4—C7	121.2 (3)	H12A—C12—H12B	107.4
C5—C4—C7	122.1 (3)	C14—C13—C18	116.5 (3)
C6—C5—C4	122.0 (3)	C14—C13—C12	120.6 (3)
C6—C5—H5	119.0	C18—C13—C12	122.8 (2)
C4—C5—H5	119.0	C15—C14—C13	121.5 (3)
C5—C6—C1	121.7 (3)	C15—C14—H14	119.3
C5—C6—H6	119.1	C13—C14—H14	119.3
C1—C6—H6	119.1	C14—C15—C16	122.0 (3)
C4—C7—H7A	109.5	C14—C15—H15	119.0
C4—C7—H7B	109.5	C16—C15—H15	119.0
H7A—C7—H7B	109.5	C17—C16—C15	117.1 (3)
C4—C7—H7C	109.5	C17—C16—C19	121.2 (3)
H7A—C7—H7C	109.5	C15—C16—C19	121.7 (3)
H7B—C7—H7C	109.5	C16—C17—C18	120.8 (3)
C9—C8—C1	111.4 (2)	C16—C17—H17	119.6
C9—C8—C10	110.0 (2)	C18—C17—H17	119.6
C1—C8—C10	113.4 (2)	C13—C18—C17	122.0 (2)
C9—C8—H8	107.3	C13—C18—H18	119.0
C1—C8—H8	107.3	C17—C18—H18	119.0
C10—C8—H8	107.3	C16—C19—H19A	109.5
O3—C9—O2	122.2 (2)	C16—C19—H19B	109.5
O3—C9—C8	123.4 (2)	H19A—C19—H19B	109.5
O2—C9—C8	114.4 (2)	C16—C19—H19C	109.5
C11—C10—C8	113.4 (2)	H19A—C19—H19C	109.5
C11—C10—H10A	108.9	H19B—C19—H19C	109.5
C8—C10—H10A	108.9	C9—O2—H2A	109.5
C11—C10—H10B	108.9		

### Hydrogen-bond geometry (Å, °)

**Table:**

*D*—H···*A*	*D*—H	H···*A*	*D*···*A*	*D*—H···*A*
C17—H17···O1^i^	0.93	2.50	3.418 (4)	169
C15—H15···O2^ii^	0.93	2.56	3.452 (4)	160
O2—H2A···O3^iii^	0.82	1.83	2.638 (2)	169

Symmetry codes: (i) −*x*, −*y*, −*z*; (ii) −*x*, *y*+1/2, −*z*+1/2; (iii) −*x*+1, −*y*, −*z*+1.
